# Hydrogen Gas Inhalation Alleviates Airway Inflammation and Oxidative Stress on Ovalbumin-Induced Asthmatic BALB/c Mouse Model

**DOI:** 10.3390/antiox13111328

**Published:** 2024-10-30

**Authors:** Wenjing He, Md. Habibur Rahman, Johny Bajgai, Sofian Abdul-Nasir, Chaodeng Mo, Hui Ma, Seong Hoon Goh, Kim Bomi, Hyeran Jung, Cheol-Su Kim, Hyungdon Lee, Kyu-Jae Lee

**Affiliations:** 1 Department of Convergence Medicine, Wonju College of Medicine, Yonsei University, Wonju 26426, Republic of Korea; hwjing20@gmail.com (W.H.); globaldreamer1990@gmail.com (M.H.R.); johnybajgai@yonsei.ac.kr (J.B.); abdulnasirsofian62@gmail.com (S.A.-N.); chaodengmo@gmail.com (C.M.); mahui56@yonsei.ac.kr (H.M.); forget419@yonsei.ac.kr (S.H.G.); kimbomi9090@gmail.com (K.B.); mdjuliet@nate.com (H.J.); cs-kim@yonsei.ac.kr (C.-S.K.); 2 Department of Global Medical Science, Wonju College of Medicine, Yonsei University, Wonju 26426, Republic of Korea; 3 Department of Internal Medicine, College of Medicine, Hallym University, Chuncheon Sacred Heart Hospital, Chuncheon 24253, Republic of Korea

**Keywords:** airway inflammation, hydrogen gas, oxidative stress, cytokines, immunoglobulin E

## Abstract

Airway inflammatory diseases, such as asthma, are a global public health concern owing to their chronic inflammatory effects on the respiratory mucosa. Molecular hydrogen (H_2_) has recently been recognized for its antioxidant and anti-inflammatory properties. In this study, we examined the therapeutic potential of H_2_ in airway inflammation using an ovalbumin (OVA)-induced BALB/c mouse model of allergic asthma. Female BALB/c mice were sensitized and challenged with OVA to induce airway inflammation, and 30 mice were randomly divided into five groups: NT (non-treatment), HTC (3% H_2_ treatment only), NC (negative control, OVA only), PC (positive control, OVA + intranasal 1 mg/mL salbutamol 50 μL), and HT (H_2_ treatment, OVA + inhaled 3% H_2_). Various inflammatory and oxidative stress (OS)-induced markers such as white blood cells (WBCs) and their differential counts, lung histology, cytokine levels such as interleukin (IL)-4, (IL)-5, (IL)-13, interferon-gamma (IFN-γ), tumor necrosis factor-alpha (TNF-α), granulocyte-macrophage colony-stimulating factor (GM-CSF), (IL)-10, reactive oxygen species (ROS), nitric oxide (NO), glutathione peroxidase (GPx), and catalase (CAT), and total immunoglobulin E (IgE) levels were investigated. Our results showed that inhaled H_2_ significantly reduced inflammatory cell infiltration, OS markers, and pro-inflammatory cytokine expression while upregulating antioxidant enzyme activity. Furthermore, H_2_ also significantly decreased serum IgE levels, a marker of allergic inflammation. Collectively, our findings suggest that H_2_ inhalation is a promising treatment option for airway inflammation, offering a novel approach with potential clinical applications.

## 1. Introduction

Airway inflammation is a collective term used to describe a group of inflammatory diseases that affect the respiratory system, such as asthma, and poses a significant threat to public health globally [1]. In 2019, chronic respiratory disorders were the underlying cause of premature mortality in 71.1 million individuals and a contributing factor to disability in 32.4 million [2]. These diseases are characterized by chronic inflammation of the respiratory tract mucosa, which leads to structural and functional disorders. This, in turn, causes dyspnea, a cough, and shortness of breath, severely impairing patients’ quality of life. Inhaled glucocorticoids and bronchodilators [3,4] are often used to effectively manage inflammatory airway diseases [5]. However, their long-term use is limited by adverse effects and the development of drug resistance [6]. Thus, the development of novel therapeutic strategies is essential for improving the management of airway inflammation.

Asthma is a complex inflammatory condition of the lungs characterized by airway inflammation and is rich in eosinophils [7]. Immune cells, including airway epithelial cells, neutrophils, lymphocytes, and eosinophils [8], trigger several inflammatory responses upon activation, such as reactive oxygen species (ROS), nitric oxide (NO), and cytokines such as interferon-gamma (IFN-γ), interleukin (IL)-4, tumor necrosis factor α (TNF-α), (IL)-10, granulocyte-macrophage colony-stimulating factor (GM-CSF), IL-5, and IL-13 [9]. It has been represented that both inflammation and oxidative stress (OS) significantly contribute to the pathophysiology of allergic asthma [10]. The overexpression of inflammatory cells may enhance ROS generation, which contributes to epithelial damage [11,12]. In turn, this triggers inflammatory pathways, leading to cytokine transmission [13]. Additionally, the pathophysiology of airway inflammation has been linked to specific functions [14], which regulate inflammatory cell infiltration and mucus production in lung disorders [15]. Radical scavengers or antioxidants may be beneficial in counterbalancing ROS formation [16]. It is well known that glutathione peroxidase (GPx) and catalase (CAT) are essential for the metabolism of oxygen. Among inflammatory cells, eosinophils invariably respond to an increase in the activity of major antioxidant enzymes when stimulated [17]. Moreover, eosinophils can present antigens to T cells, which helps trigger inflammatory cascade responses even further [18]. Exposure to allergens stimulates Th2 cytokines (IL-4, IL-5, and IL-13), which then activate eosinophils and increase the production of immunoglobulin E (IgE) [19].

Over the past few years, molecular hydrogen (H_2_) has gradually gained interest as a potential new treatment option [20,21], and the anti-inflammatory and antioxidant properties of H_2_ in a range of diseases have been demonstrated in several studies [22,23,24]. H_2_ has been described as an antioxidant that suppresses the amounts of peroxynitrite (ONOO^−^) and hydroxyl radicals (^•^OH) to protect cells from OS [25]. Moreover, H_2_ has been shown to exert anti-inflammatory effects, notably through its ability to downregulate pro-inflammatory cytokines such as IL-1β, IL-6, and TNF-α [25]. By inhibiting these key inflammatory mediators, H_2_ reduces OS and inflammation, thereby offering therapeutic benefits. Specifically, its role in alleviating airway inflammation has been demonstrated, suggesting its potential application in inflammatory respiratory conditions [26]. According to Zhang et al. [27], H_2_ inhalation decreased lung resistance in asthmatic mice, reversing inflammatory infiltration and goblet cell hyperplasia. It also reduced total cells, eosinophils, and lymphocytes in bronchoalveolar lavage fluid; decreased TNF-α, IL-4, IL-13, and C-X-C motif chemokine ligand 15 levels; and decreased upregulated superoxide dismutase activity, while attenuating increased malondialdehyde and myeloperoxidase levels.

Significant advances have been made in the understanding of the therapeutic potential of various diseases. However, their role in airway inflammation remains poorly understood. Therefore, this study aims to evaluate the anti-inflammatory and antioxidant properties of H_2_ in Ovalbumin (OVA)-induced airway inflammatory animal models and explore its potential as a therapeutic agent, with the goal of advancing its clinical potential applications in treating inflammatory respiratory conditions.

## 2. Materials and Methods

### 2.1. Animals

Female BALB/c mice (8–10 weeks), with an average weight of 20 ± 2 g, were obtained from Orient Bio Inc. (Seongnam, Republic of Korea). Prior to the start of the experiment, the mice were kept in a specific pathogen-free facility under controlled conditions, including a 12-h light/dark cycle, a temperature of 22 ± 2 °C, and humidity levels ranging between 40% to 60%. The mice had unrestricted access to water and standard laboratory chow. All experimental procedures followed the guidelines set by the National Animal Welfare Law of the Republic of Korea, and the protocol was approved by the Institutional Animal Care and Use Committee of Yonsei University Wonju College of Medicine (approval number: YWC-230530-1).

### 2.2. Sensitization and Challenge of Airway Inflammation with OVA

**Figure 1:**
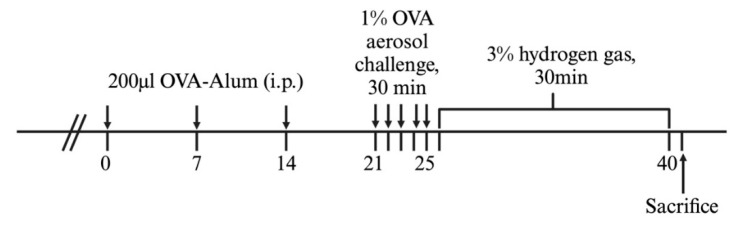
Animal experimental procedures.

OVA from Sigma-Aldrich (St. Louis, MO, USA) was used for both sensitization and challenge to establish mouse models of airway inflammation. A total of 30 mice were randomly divided into five groups, each consisting of six mice: NT (non-treatment), HTC (3% H_2_ treatment only), NC (negative control with OVA only), PC (positive control with OVA + intranasal 1 mg/mL salbutamol 50 μL), and HT (OVA + 3% H_2_ inhalation). On days 0, 7, and 14, mice were sensitized through intraperitoneal injections of OVA mixed with alum. Subsequently, mice were exposed to aerosolized 1% OVA or phosphate-buffered saline (PBS) for 30 min daily from days 21 to 25. Mice in the HTC and HT groups received 3% H_2_ gas inhalation for 30 min over 15 consecutive days following the OVA or PBS challenge (Figure 1).

### 2.3. Hydrogen Gas Administration

The hydrogen-generating device, which was designed and supplied by GOOTZ Co., Ltd. (Yangju-si, Gyeonggi-do, Republic of Korea), produced H_2_ gas at a saturation concentration of 3%. A transparent sealed chamber measuring 32 × 22 × 14 cm^3^ (length × width × height) was used for all of the experimental treatments. During each experiment, the concentration of H_2_ gas inside the chamber was monitored using a combustible gas detector (Cosmos, Japan).

### 2.4. White Blood Cell (WBC) and Its Differential Counts

Blood samples were collected from the retro-orbital plexus and immediately placed in tubes containing ethylenediaminetetraacetic acid (EDTA) as an anticoagulant. The total WBC count and the differential counts of neutrophils, lymphocytes, and eosinophils were determined using an automated blood analyzer (HEMAVET HV950 FS; Drew Scientific Inc., Boston, MA, USA).

### 2.5. Lung Histology

For morphological assessment, the left lung lobe was excised and fixed overnight in 10% formaldehyde. After fixation, the tissue samples were dehydrated, embedded in paraffin wax, and sectioned into 4–6 µm slices using a microtome. The sections were deparaffinized and stained with hematoxylin and eosin (H&E). The right lung was preserved at −80 °C for further additional studies. The degree of peribronchial inflammation was measured using H&E-stained lung sections.

### 2.6. Detection of Inflammation Cytokines in Serum

Following the manufacturer’s instructions, a bead array Suspension Multiplex Kit (Bio-Rad, San Diego, CA, USA) was used to analyze the inflammatory cytokines, including IL-4, IL-5, IL-13, IFN-γ, TNF-α, GM-CSF, and IL-10 in the serum sample. Cytokine analysis was performed using the Luminex 200 Bio-Plex system, and raw data were analyzed using a five-parameter logistic method.

### 2.7. Total ROS and NO Level Measurement in the Serum

The total ROS in the serum was quantified using the oxidation of 2-4-dichlorodihydrofluorescein diacetate (DCFH-DA) (Abcam, Cambridge, MA, USA) following established protocols. Serum samples (10 µL) were added to a 96-well plate, followed by 100 µL of 20 µM DCFH-DA. The plate was incubated in the dark for 30 min and fluorescence was measured using a DTX-880 multimode microplate reader (Beckman Coulter Inc., Fullerton, CA, USA) at an excitation wavelength of 488 nm and emission wavelength of 525 nm [28].

Additionally, serum NO levels were evaluated using the Griess reagent (Biomax Co., Ltd., Seoul, Republic of Korea) according to the manufacturer’s guidelines. Nitrite levels were determined by mixing 50 µL of serum with 50 µL of Griess Reagent I and 50 µL of Griess Reagent II. The samples were incubated for 10 min in the dark at room temperature and optical density (OD) was measured at 540 nm using a SpectraMax^®^ ABS Plus (Molecular Devices, San Jose, CA, USA).

### 2.8. GPx and CAT Enzyme Activity Level Estimation

The intracellular levels of endogenous antioxidant enzymes (CAT and GPx) were assessed using a Cayman kit (Cayman, Ann Arbor, MI, USA) according to the manufacturer’s guidelines. Briefly, 78 µL of samples (10 µL from stock and 68-µL assay buffer) for the CAT assay and 50 µL of samples (10 µL from stock and 40-µL assay buffer) for the GPx assay were added to a 96-well microplate, and the plates were incubated for 30 min. The optical densities of CAT (570 nm) and GPx (340 nm) were measured using SpectraMax^®^ ABS Plus (Molecular Devices, San Jose, CA, USA). CAT and GPx activities are expressed in nmol/min/mL and mU/mL, respectively.

### 2.9. Total IgE Level in Serum

Blood samples were collected from the retro-orbital plexus of mice. Serum was obtained by centrifuging the samples at 13,000 rpm for 5 min and subsequently stored at −80 °C until further analysis. The total serum IgE levels were measured using a mouse IgE ELISA kit (Cusabio, Wuhan, China), following the manufacturer’s instructions. The OD was read at 450 nm using a SpectraMax^®^ ABS Plus microplate reader (Molecular Devices, San Jose, CA, USA).

### 2.10. Statistical Analysis

Data are expressed as the mean ± standard deviation (SD). Statistical analysis was performed using a one-way analysis of variance (ANOVA), followed by a post-hoc multiple comparison test using GraphPad Prism (version 10.0; GraphPad, La Jolla, CA, USA). Statistical significance was set at *p* < 0.05.

## 3. Results

### 3.1. Effects of H_2_ on Body and Lung Weight in the Airway Inflammatory Mouse Model

**Figure 2:**
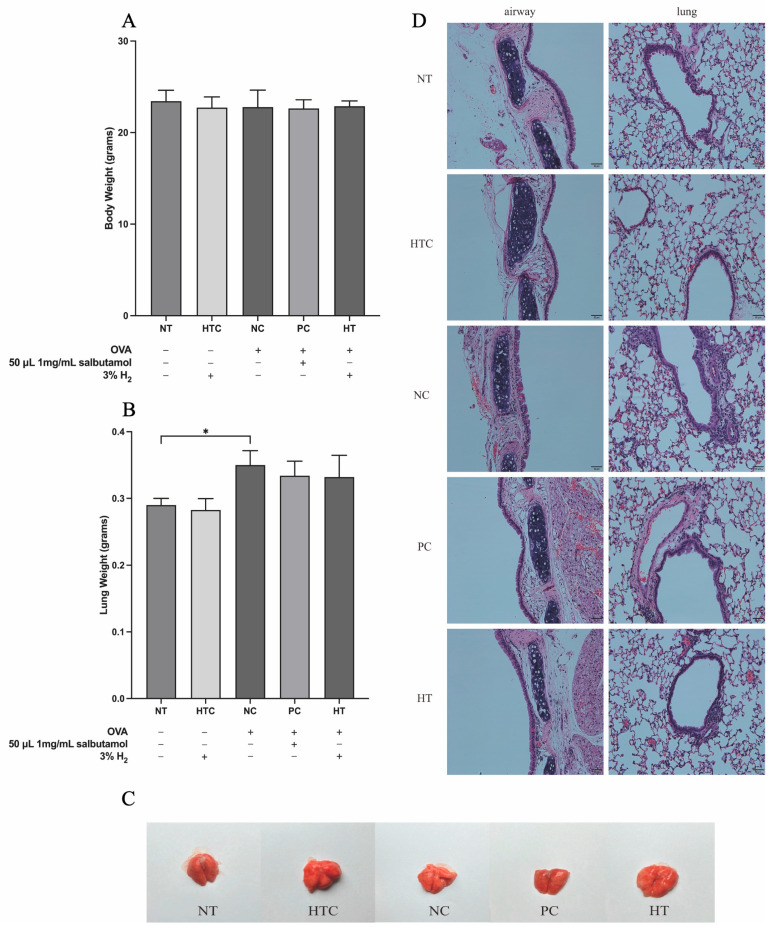
Effects of H_2_ gas inhalation on body and lung weight characteristics. (**A**) Mice body weight, (**B**) lung weight, (**C**) appearance of lung organs, (**D**) lung tissue histological analysis with H&E staining, highlighting morphological features. Data are expressed as mean ± SD (*n* = 5). Statistical significance is indicated as * *p* < 0.05.

To evaluate the effects of 3% H_2_ gas inhalation on OVA-induced inflammatory airway conditions, body and lung weights were recorded in mice. No significant differences in body or lung weights were observed between the NT and NC groups (Figure 2A,B). However, the lung weight was significantly higher in the NC group than in the NT group (*p* < 0.05), as shown in Figure 2B. Furthermore, no visible pathological changes were detected in the lungs across the five experimental groups (Figure 2C).

### 3.2. Effects of H_2_ on the Inhibition of Inflammatory Cell Infiltration in the Airway Inflammatory Mouse Model

**Figure 3:**
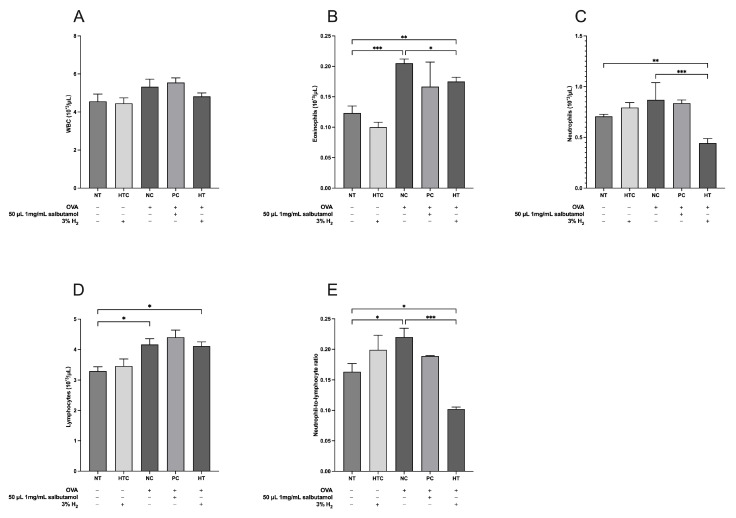
Inhalation of H_2_ gas regulates the inflammatory cell levels in the blood of allergic mouse models. (**A**) Total WBCs, (**B**) eosinophils, (**C**) neutrophils, (**D**) lymphocytes, and (**E**) neutrophil-to-lymphocyte ratio. Data are expressed as mean ± SD (*n* = 5). Statistical significance is indicated as * *p* < 0.05; ** *p* < 0.01; *** *p* < 0.001.

A key characteristic feature of asthma is the infiltration and activation of immune cells [29]. To evaluate the effects of 3% H_2_ inhalation, we analyzed lung histology (Figure 2D) and quantified inflammatory cell numbers (Figure 3). Mice in the NT and HTC groups had normal lung and airway structures, with minimal inflammatory cells. Conversely, the NC group exhibited inflammatory cell infiltration, thickened alveolar walls, and structural airway abnormalities (Figure 2D). Treatment with salbutamol and H_2_ in the PC and HT groups revealed a reduction in inflammatory cells in the lungs (Figure 2D).

WBCs play a vital role in both innate and adaptive responses [30], with eosinophils, neutrophils, and lymphocytes as hallmarks of allergic inflammation [29]. The neutrophil-to-lymphocyte ratio (NLR) is a parameter that integrates neutrophils, representing innate inflammation and lymphocytes, which act as regulators of allergic inflammation. It has been proposed that blood NLR could serve as an indicator of the body’s overall inflammation and stress levels [31]. WBC count analysis revealed significant differences between the experimental groups (Figure 3). Specifically, eosinophil levels in the NC group were significantly higher than those in the NT (*p* < 0.001; Figure 3B) and HT groups (*p* < 0.05; Figure 3B). The HT group was significantly higher than the NT group (*p* < 0.01; Figure 3B). Additionally, the NC group exhibited a notable decrease in neutrophils compared to the HT group (*p* < 0.001; Figure 3D). Lymphocyte counts were significantly higher in the NC group and HT group than in the NT group (*p* < 0.05; Figure 3C). Our results also demonstrated a significant decline in the NLR in the HT group compared to the NT group (*p* < 0.05; Figure 3E) and the NC group (*p* < 0.001; Figure 3E). These results indicate that a 3% H_2_ inhalation slightly reduces the infiltration of inflammatory cells in the lungs of OVA-induced mice.

### 3.3. Effects of H_2_ Inhalation on the Level of Inflammatory Cytokines in the Airway Inflammatory Mouse Model

**Figure 4:**
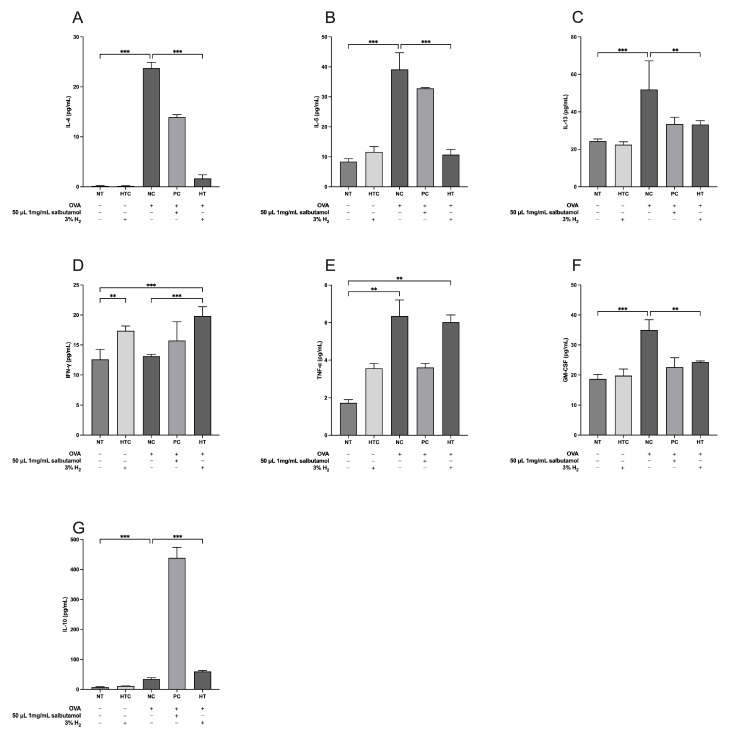
Inhalation of H_2_ gas modulates the expression of serum cytokine levels in an OVA-induced airway inflammatory mouse model. (**A**) IL-4, (**B**) IL-5, (**C**) IL-13, (**D**) IFN-γ, (**E**) TNF-α, (**F**) GM-CSF, and (**G**) IL-10. Data are expressed as mean ± SD (*n* = 5). Statistical significance is indicated as ** *p* < 0.01; *** *p* < 0.001.

To evaluate the effects of inhalation of 3% H_2_ on serum inflammatory cytokines, we measured IL-4, IL-5, IL-13, IFN-γ, TNF-α, GM-CSF, and IL-10 (Figure 4). Our results indicated that the administration of H_2_ gas significantly elevated IFN-γ (*p* < 0.001; Figure 4D) and IL-10 (*p* < 0.001; Figure 4G) levels in the HT group compared to the NC group. Conversely, the levels of IL-4 (*p* < 0.001; Figure 4A), IL-5 (*p* < 0.001; Figure 4B), IL-13 (*p* < 0.01; Figure 4C), and GM-CSF (*p* < 0.01; Figure 4F) were significantly lower in the HT group than in the NC group. Notably, no significant difference was observed in the serum TNF-α levels (Figure 4E) compared to the NC group. Collectively, these results show that the inhalation of 3% H_2_ gas effectively attenuates serum inflammation in OVA-induced airway inflammatory mouse models.

### 3.4. Effects of H_2_ in Preventing Oxidative Damage in the Airway Inflammatory Mouse Model

**Figure 5:**
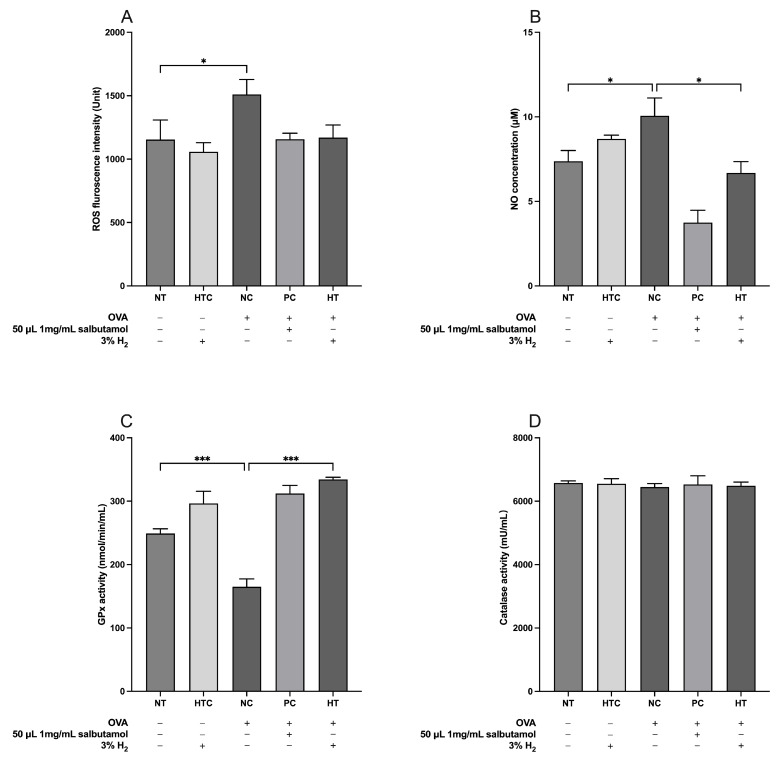
Inhalation of H_2_ gas effects on redox markers in an airway inflammatory mouse model. Serum levels of (**A**) ROS, (**B**) NO, (**C**) GPx, and (**D**) CAT levels. Data are expressed as mean ± SD (*n* = 5). Statistical significance is indicated as * *p* < 0.05; *** *p* < 0.001.

OVA-induced allergic inflammation is associated with elevated OS in mice. We assessed serum levels of ROS, NO, GPx, and CAT to evaluate the redox effects of H_2_ inhalation in the airway inflammation model. As shown in Figure 5, the ROS and NO levels significantly increased in the NC group (*p* < 0.05) compared with the NT group. Our results revealed that NO (*p* < 0.05; Figure 5B) levels were reduced in the HT group compared with those in the NC group. Additionally, GPx activity was significantly lower in the NC group than in the NT group (*p* < 0.001; Figure 5C). Contrarily, treatment with 3% H_2_ significantly increased the GPx activity in the HT group compared to that in the NC group (*p* < 0.001; Figure 5C), suggesting its protective role against oxidative damage in airway inflammation.

### 3.5. Effects of H_2_ Inhalation on the Total IgE Levels in the Airway Inflammatory Mouse Model

**Figure 6:**
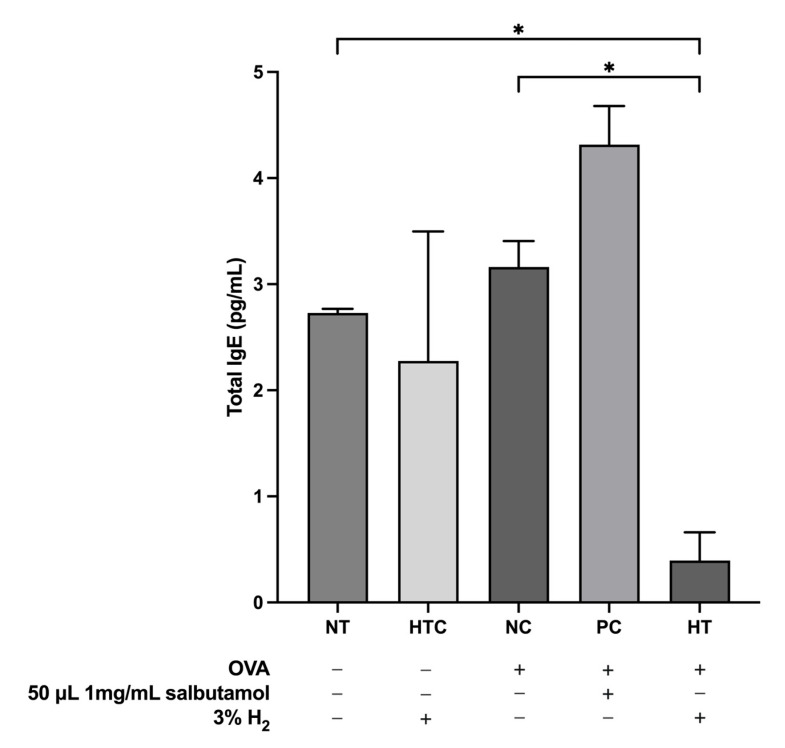
Inhalation of H_2_ gas reduced the total IgE level in the serum of the OVA-induced airway inflammatory mouse model. Data are expressed as mean ± SD (*n* = 5). Statistical significance is indicated as * p < 0.05.

Total IgE production is associated with allergic inflammation. In this study, we evaluated the effect of inhaled 3% H_2_ gas on the serum levels of total IgE. Our results demonstrated a significant decline in serum total IgE levels in the HT group compared to those in the NC group (*p* < 0.05; Figure 6). This reduction suggests that inhalation of H_2_ gas might effectively mitigate allergic responses in a mouse model by lowering IgE levels, which are indicators of the severity of allergic inflammation.

## 4. Discussion

In the present study, we evaluated the anti-inflammatory and antioxidant effects of 3% H_2_ gas inhalation in an OVA-induced allergic asthmatic mouse model. Our findings indicate that H_2_ exhibits significant anti-asthmatic properties by effectively reducing inflammatory cell infiltration, decreasing NO levels, upregulating the activity of the antioxidant enzyme GPx, and enhancing the expression of anti-inflammatory cytokines such as IFN-γ and IL-10. Additionally, H_2_ treatment significantly decreased the production of pro-inflammatory cytokines, including IL-4, IL-5, IL-13, GM-CSF, and total IgE.

The two forms of asthma phenotypes are type 2-low, characterized by non-eosinophilic inflammation, neutrophilic involvement, metabolic responses, and type 2-high IL-5 and IL-13, which are primarily eosinophilic and pathologically driven by Th2 cells that produce IL-4. The type 2-high phenotype is observed in the majority of asthmatics with moderate to severe disease [32,33], and asthma was long considered the definitive example of a Th2-mediated airway disease, supported by evidence from mouse models [29]. Type 2 (Th2) inflammation of the airways has been shown to be a major molecular mechanism of asthma in mice [33]. Allergic airway inflammation in most asthmatic mouse models is induced by one or more intraperitoneal injections of a protein allergen, typically OVA, combined with aluminum hydroxide (alum), which acts as a Th2-skewing adjuvant [34]. Mouse models of OVA-induced allergic airway inflammation show several characteristics of human asthma, including increased serum IgE concentrations, eosinophil infiltration, and increased levels of Th2 cytokines (IL-4, IL-5, and IL-13) [35,36]. Similar results were observed in our mouse model, which showed increased levels of cytokines, IgE, and serum eosinophil infiltration after OVA induction.

An essential determinant of inflammation intensity is the number of inflammatory cells. The total WBC count, which includes eosinophils, neutrophils, and lymphocytes, is a recognized indicator of the systemic inflammatory response and can be used as a biomarker to investigate the relationship between systemic inflammation and lung function in population-based research [37,38]. Recently, numerous inflammatory markers, such as the WBC count, eosinophil count, and NLR, have shown promise as indicators of asthma [39,40]. Eosinophils are associated with the development of OVA-induced airway inflammation, where they contribute to injuries, obstructions, and hyperresponsiveness of the bronchi, either by themselves or through their interactions with different pro-inflammatory pathways [41,42,43,44]. Jin et al. discovered that H_2_ gas has preventive effects against allergic diseases by reducing the number of eosinophils in the blood [45]. Neutrophils are cells that could play a significant role in causing respiratory symptoms, which are likely to be linked to hyperresponsiveness [46,47]. In our study, we observed that H_2_ inhalation reduced neutrophils in the OVA-induced asthmatic BALB/c mouse model. This finding is consistent with the results reported by Xiao et al. [48]. Xie and colleagues found that H_2_ treatment reduced lipopolysaccharide (LPS)-induced neutrophil recruitment into the lungs, thereby attenuating lung inflammation [49]. Th2 lymphocytes are essential in producing a range of interleukins (IL-4, IL-5, IL-13) and GM-CSF, which facilitate communication with other cells and maintain inflammation [50]. NLR is a biomarker of generalized inflammation in pulmonology and has the potential to yield diagnostically relevant biomarkers that are therapeutically significant [51,52]. Asseri and colleagues found that the NLR was significantly higher during acute asthma exacerbations in children compared to stable asthma, but it had no significant effect in adults [39]. The NLR was significantly increased in our study and clearly decreased after inhaling 3% H_2_; it could reflect neutrophil-associated systemic inflammation [53]. NLR is predominantly used in clinical research, with few reports focusing on its application in mice with allergic asthma [54,55]. Clinical trials indicate a significant difference in the lymphocytes and neutrophils among adult animals. Mouse blood contains a higher proportion of lymphocytes (75–90%) and a lower proportion of neutrophils (10–25%), whereas human blood has a greater concentration of neutrophils (50–70%) and lymphocytes (30–50%) [56]. This is the first time that NLR has been reported in asthmatic mice with allergic airway inflammation, to the best of our knowledge, but their role in asthma assessment remains limited.

Understanding how inflammatory mediators function in chronic inflammatory processes is essential as they seem to dictate the characteristics of the inflammatory response by guiding the targeted recruitment and activation of inflammatory cells and their sustained presence in the lung [57]. IFN-γ enhances the action of TNF-α and triggers inflammation-induced NO, leading to tissue damage at the inflammatory site. Effective inhibition of TNF-α is very advantageous in managing chronic inflammatory disorders [58]. IL-10, a cytokine with anti-inflammatory properties, affects several immune cells implicated in allergic asthma. It inhibits eosinophilic function, stimulates IFN-γ production, and favors B-cell expansion from IgE [59]. Our data revealed that after the administration of 3% H_2_ gas that promotes the release of IFN-γ and IL-10, notwithstanding that no significant changes were observed in TNF-α concentration, there was a significant reduction in NO levels. Allergic airway inflammation is significantly influenced by the Th2-mediated cytokines IL-4, IL-5, and IL-13, which act as cellular targets for a range of responses. IL-4 and IL-5 are key drivers of the asthmatic eosinophilic airway inflammation type 2 pathway [41,60,61]. Likewise, IL-4 is primarily responsible for allergic inflammation and increased mucus production, which facilitates the differentiation of naïve T-helper cells into Th2 cells, contributing to the advancement of allergic diseases [62] and promoting B cell maturation and the transition to IgE [63]. Additionally, IL-5 is associated with the regulation (induction, survival, and maturation) of basophils and eosinophils. Furthermore, IL-13 has been linked to eosinophilia, where it functions in conjunction with IL-5 to facilitate eosinophil activation and migration [64]. However, in allergic inflammatory mouse models, GM-CSF inherently stimulates eosinophil build-up, thereby playing a crucial role in the development of allergic disorders [65]. Based on our findings, H_2_ was significantly attributed to its anti-inflammatory and anti-asthmatic properties. Interestingly, OVA exposure has been observed to elevate IgE levels, indicating an allergic or chronic inflammatory response in the lungs [66]. Interleukins, particularly IL-4 and IL-5, are responsible for the synthesis of IgE, stimulating eosinophilia, modulating eosinophil function, and enhancing the proliferation of mucosal-type mast cells [67]; however, the inhalation of H_2_ has been shown to reduce IgE levels and inhibit inflammatory responses.

Additionally, the oxidative damage caused by an imbalance in the redox state of the airways leads to the inflammatory response, remodeling of the airways, and hyperresponsiveness of the airways [68,69,70]. Studies demonstrated that OVA-induced airways in animals exhibit elevated levels of ROS including hydrogen peroxide (H_2_O_2_), superoxide anion (O_2_^−^), and ^•^OH generated by the enzyme nicotinamide adenine dinucleotide phosphate (NADPH) oxidase that may influence the vasculature of the airways, induce mucus production and bronchoconstriction, and increase reactivity of the airways [71,72]. It has been demonstrated that H_2_ mitigates allergic airway irritation by decreasing ROS levels [73]. Airway inflammation is largely influenced by NO and its byproducts, although the metabolites remain unclear [74]. Although NO and its derivatives can be bronchoprotective [75,76], high levels of NO and reactive nitrogen species can also lead to cellular destruction. Since Th2 cells are responsible for eosinophilic inflammation, it is known that asthmatic airways have increased NO levels. These elevated NO levels lead to mucus production, plasma exudation, and hyperemia and indirectly enhance Th2 cell proliferation [76,77]. GPx and CAT are vital antioxidant enzymes in the lungs [78]. GPx is essential for the upregulation of pro-inflammatory mediators during the development of allergic asthma [79]. GPx activity is markedly decreased in asthmatic patients [80]. Liang et al. [81] showed that the application of antioxidant enzymes may significantly reduce oxidative damage and airway inflammation by reducing ROS formation and increasing the activity of GPx and CAT. However, CAT has been shown to reduce OS by augmenting gene and enzyme activity [80]. Furthermore, You et al. [82] investigated that H_2_ gas may protect airway epithelial cells from OS by suppressing the elevation of ROS and NO levels and boosting the activity of antioxidant enzymes, such as CAT and GPx, which might result in a potential respiratory condition intervention. Our study revealed that H_2_ gas increased GPx activity and decreased the NO levels in asthmatic mice. However, the impact of hydrogen gas treatment on ROS and CAT was found to be normal, indicating that the inhalation of H_2_ gas could protect against OS.

H_2_ is abundant in nature and is both odorless and colorless [83]. Owing to its small molecular weight, H_2_ can easily infiltrate a cell and access many organelles, containing the nucleus and cytoplasm, to perform its biological functions, and is recognized for leaving no remaining residues in the body’s metabolism [84,85]. Moreover, inhalation was the most convenient and widely used method in the early reports on the application of H_2_ gas [86]. Previous studies have indicated that H_2_ inhalation significantly mitigates oxidative damage in 1–3% of instances [87]. In this study, we used an allergic asthmatic mouse model, and the results demonstrated that treating airway inflammation with 3% H_2_ is safe, well tolerated, and free of adverse effects.

This study assessed the serum levels of cytokines and redox reactions in an allergic asthma mouse model. However, due to the complex mechanisms of asthma, further research is needed on H_2_ therapy for other asthma phenotypes, such as house dust mite (HDM)-induced neutrophilic asthma. Additionally, the correlations between animal models and patients are not yet fully understood. Further clinical trials are required to determine the therapeutic effects of H_2_ for respiratory inflammation in humans.

## 5. Conclusions

In conclusion, the results of this study provide compelling evidence of the potential therapeutic effects of H_2_ in allergic airway inflammation. A reduction in inflammatory cell infiltration, attenuation of airway inflammation, and suppression of IgE production were observed. This study supports the idea that H_2_ could serve as an alternative or additional treatment for allergic asthma and related diseases.

## Data Availability

All of the data are contained within the article.

## List of Abbreviations

^•^OH	Hydroxyl radicals
ANOVA	One-way analysis of variance
CAT	Catalase
DCFH-DA	2-4-dichlorodihydrofluorescein diacetate
EDTA	Ethylenediaminetetraacetic acid
GM-CSF	Granulocyte-macrophage colony-stimulating factor
GPx	Glutathione peroxidase
H&E	Hematoxylin and eosin
H₂	Hydrogen
H₂O₂	Hydrogen peroxide
HDM	House dust mite
IFN-γ	Interferon gamma
IgE	Immunoglobulin E
IL	Interleukin
LPS	Lipopolysaccharide
NADPH	Nicotinamide adenine dinucleotide phosphate
NLR	Neutrophil-to-lymphocyte ratio
NO	Nitric oxide
O₂⁻	Superoxide anion
OD	Optical density
ONOO^−^	Peroxynitrite
OS	Oxidative stress
OVA	Ovalbumin
ROS	Reactive oxygen species
SD	Standard deviation
TNF-α	Tumor necrosis factor α
WBC	White blood cell
